# Certain Inequalities Involving Generalized Erdélyi-Kober Fractional *q*-Integral Operators

**DOI:** 10.1155/2014/174126

**Published:** 2014-09-11

**Authors:** Praveen Agarwal, Soheil Salahshour, Sotiris K. Ntouyas, Jessada Tariboon

**Affiliations:** ^1^Department of Mathematics, Anand International College of Engineering, Jaipur 303012, India; ^2^Department of Computer Engineering, Mashhad Branch, Islamic Azad University, Mashhad, Iran; ^3^Department of Mathematics, University of Ioannina, 451 10 Ioannina, Greece; ^4^Nonlinear Dynamic Analysis Research Center, Department of Mathematics, Faculty of Applied Science, King Mongkut's University of Technology North Bangkok, Bangkok 10800, Thailand

## Abstract

In recent years, a remarkably large number of inequalities involving the fractional *q*-integral operators have been investigated in the literature by many authors. Here, we aim to present some new fractional integral inequalities involving generalized Erdélyi-Kober fractional *q*-integral operator due to Gaulué, whose special cases are shown to yield corresponding inequalities associated with Kober type fractional *q*-integral operators. The cases of synchronous functions as well as of functions bounded by integrable functions are considered.

## 1. Introduction

Let us start by considering the following functional (see [1]):

(1) T(f,g,p,q) =∫abq(x)dx∫abp(x)f(x)g(x)dx  +∫abp(x)dx∫abq(x)f(x)g(x)dx  −(∫abq(x)f(x)dx)(∫abp(x)g(x)dx)  −(∫abp(x)f(x)dx)(∫abq(x)g(x)dx),

where *f*, *g* : [*a*, *b*] → *R* are two integrable functions on [*a*, *b*] and *p*(*x*) and *q*(*x*) are positive integrable functions on [*a*, *b*]. If *f* and *g* are* synchronous* on [*a*, *b*], that is,

(2) $$\begin{array}{c} \left(f\left(x\right)-f\left(y\right)\right)\left(g\left(x\right)-g\left(y\right)\right)\geq 0, \end{array}$$

for any *x*, *y* ∈ [*a*, *b*], then we have (see, e.g., [2, 3])

(3) $$\begin{array}{c} T\left(f,g,p,q\right)\geq 0. \end{array}$$

The inequality in (2) is reversed if *f* and *g* are* asynchronous* on [*a*, *b*]; that is,

(4) $$\begin{array}{c} \left(f\left(x\right)-f\left(y\right)\right)\left(g\left(x\right)-g\left(y\right)\right)\leq 0, \end{array}$$

for any *x*, *y* ∈ [*a*, *b*]. If *p*(*x*) = *q*(*x*) for any *x*, *y* ∈ [*a*, *b*], we get the Chebyshev inequality (see [1]). Ostrowski [4] established the following generalization of the Chebyshev inequality.

If *f* and *g* are two differentiable and synchronous functions on [*a*, *b*] and *p* is a positive integrable function on [*a*, *b*] with |*f*′(*x*)|≥*m* and |*g*′(*x*)|≥*r* for *x* ∈ [*a*, *b*], then we have

(5) T(f,g,p)=T(f,g,p,p)≥mrT(x−a,x−a,p)≥0.

If *f* and *g* are asynchronous on [*a*, *b*], then we have

(6) T(f,g,p)≤mrT(x−a,x−a,p)≤0.

If *f* and *g* are two differentiable functions on [*a*, *b*] with |*f*′(*x*)|≤*M* and |*g*′(*x*)|≤*R* for *x* ∈ [*a*, *b*] and *p* is a positive integrable function on [*a*, *b*], then we have

(7) |T(f,g,p)|≤MRT(x−a,x−a,p)≤0.

Here, it is worth mentioning that the functional (1) has attracted many researchers' attention mainly due to diverse applications in numerical quadrature, transform theory, probability, and statistical problems. Among those applications, the functional (1) has also been employed to yield a number of integral inequalities (see, e.g., [5–11]).

The study of the fractional integral and fractional *q*-integral inequalities has been of great importance due to the fundamental role in the theory of differential equations. In recent years, a number of researchers have done deep study, that is, the properties, applications, and different extensions of various fractional *q*-integral operators (see, e.g., [12–16]).

The purpose of this paper is to find *q*-calculus analogs of some classical integral inequalities. In particular, we will find *q*-generalizations of the Chebyshev integral inequalities by using the generalized Erdélyi-Kober fractional *q*-integral operator introduced by Galué [17]. The main objective of this paper is to present some new fractional *q*-integral inequalities involving the generalized Erdélyi-Kober fractional *q*-integral operator. We consider the case of synchronous functions as well as the case of functions bounded by integrable functions. Some of the known and new results are as follows, as special cases of our main findings. We emphasize that the results derived in this paper are more generalized results rather than similar published results because we established all results by using the generalized Erdélyi-Kober fractional *q*-integral operator. Our results are general in character and give some contributions to the theory *q*-integral inequalities and fractional calculus.

## 2. Preliminaries

In the sequel, we required the following well-known results to establish our main results in the present paper. The *q*-*shifted factorial *(*a*; *q*)_*n*_ is defined by

(8) $$\begin{array}{c} {\left(a;q\right)}_{n}:=\{\begin{array}{cc} 1, & \left(n=0\right)\\ \overset{n-1}{\underset{k=0}{\prod }}\left(1-aq^{k}\right), & \left(n\in N\right), \end{array} \end{array}$$

where *a*, *q* ∈ *C* and it is assumed that *a* ≠ *q* ^−*m*^  (*m* ∈ *N*_0_).

The *q*-*shifted factorial* for negative subscript is defined by

(9) (a;q)−n≔1(1−aq−1)(1−aq−2)⋯(1−aq−n)(n∈N0).

We also write

(10) $$\begin{array}{c} {\left(a;q\right)}_{\infty }≔\overset{\infty }{\underset{k=0}{\prod }}\left(1-aq^{k}\right)\;\left(a,q\in C;\;\;\left|q\right|<1\right). \end{array}$$

It follows from (8), (9), and (10) that

(11) $$\begin{array}{c} {\left(a;q\right)}_{n}=\frac{{\left(a;q\right)}_{\infty }}{{\left(aq^{n};q\right)}_{\infty }}\;\left(n\in Z\right), \end{array}$$

which can be extended to *n* = *α* ∈ *C* as follows:

(12) $$\begin{array}{c} {\left(a;q\right)}_{\alpha }=\frac{{\left(a;q\right)}_{\infty }}{{\left(aq^{\alpha };q\right)}_{\infty }}\;\left(\alpha \in C;\;\;\left|q\right|<1\right), \end{array}$$

where the principal value of *q* ^*α*^ is taken.

We begin by noting that F. J. Jackson was the first to develop *q*-calculus in a systematic way. For 0 < *q* < 1, the *q*-*derivative* of a continuous function *f* on [0, *b*] is defined by

(13) Dqf(t)≔dqdqtf(t)=f(t)−f(qt)(1−q)t, t∈(0,b],

and *D*_*q*_ *f*(0) = lim⁡_*t*→0_ *D*_*q*_ *f*(*t*). It is noted that

(14) $$\begin{array}{c} \underset{q\to 1}{\lim}D_{q}f\left(t\right)=\frac{d}{dt}f\left(t\right), \end{array}$$

if *f*(*t*) is differentiable.

The function *F*(*t*) is a *q*-*antiderivative* of *f*(*t*) if *D*_*q*_ *F*(*t*) = *f*(*t*). It is denoted by

(15) $$\begin{array}{c} \int f\left(t\right)d_{q}t. \end{array}$$

The* Jackson integral* of *f*(*t*) is thus defined,* formally*, by

(16) ∫f(t)dqt≔(1−q)t∑j=0∞qjf(qjt),

which can be easily generalized as follows:

(17) ∫f(t)dqg(t)=∑j=0∞f(qjt)(g(qjt)−g(qj+1t)).

Suppose that 0 < *a* < *b*. The definite *q*-integral is defined as follows:

(18) $$\begin{array}{c} \overset{b}{\underset{0}{\int }}f\left(t\right)d_{q}t:=\left(1-q\right)b\overset{\infty }{\underset{j=0}{\sum }}q^{j}f\left(q^{j}b\right), \end{array}$$

(19) ∫abf(t)dqt=∫0bf(t)dqt−∫0af(t)dqt.

A more general version of (18) is given by

(20) $$\begin{array}{c} \overset{b}{\underset{0}{\int }}f\left(t\right)d_{q}g\left(t\right)=\overset{\infty }{\underset{j=0}{\sum }}f\left(q^{j}b\right)\left(g\left(q^{j}b\right)-g\left(q^{j+1}b\right)\right). \end{array}$$

The classical Gamma function Γ(*z*) (see, e.g., [18, Section  1.1]) was found by Euler while he was trying to extend the factorial *n*! = Γ(*n* + 1)(*n* ∈ *N*_0_) to real numbers. The *q*-factorial function [*n*]_*q*_!  (*n* ∈ *N*_0_) of *n*! defined by

(21) $$\begin{array}{c} {\left[n\right]}_{q}!≔\{\begin{array}{cc} 1, & \text{if}\;\;n=0,\\ {\left[n\right]}_{q}{\left[n-1\right]}_{q}\cdots {\left[2\right]}_{q}{\left[1\right]}_{q}, & \text{if}\;\;n\in N, \end{array} \end{array}$$

can be rewritten as follows:

(22) (1−q)−n∏k=0∞(1−qk+1)(1−qk+1+n)=(q;q)∞(qn+1;q)∞(1−q)−n≔Γq(n+1) (0<q<1).

Replacing *n* by *a* − 1 in (22), Jackson [19] defined the *q*-Gamma function Γ_*q*_(*a*) by

(23) $$\begin{array}{c} \Gamma_{q}\left(a\right)≔\frac{{\left(q;q\right)}_{\infty }}{{\left(q^{a};q\right)}_{\infty }}{\left(1-q\right)}^{1-a}\;\left(0<q<1\right). \end{array}$$

The *q*-analogue of (*t* − *a*)^*n*^ is defined by the polynomial

(24) (t−a)(n)≔{1,(n=0)(t−a)(t−qa)⋯(t−qn−1a),(n∈N),=tn(at;q)n (n∈N0).

More generally, if *γ* ∈ *R*, then

(25) $$\begin{array}{c} {\left(t-a\right)}^{\left(\gamma \right)}≔t^{\gamma }\overset{\infty }{\underset{k=0}{\prod }}\frac{1-\left(a/t\right)q^{k}}{1-\left(a/t\right)q^{\gamma +k}},\;t\neq 0. \end{array}$$

**Definition 1 .**

Let *R*(*β*), *R*(*μ*) > 0 and *η* ∈ *C*. Then a generalized Erdélyi-Kober fractional integral *I*_*q*_^*α*,*β*,*η*^ for a real-valued continuous function *f*(*t*) is defined by (see, [17])

(26) Iqη,μ,β{f}(t)  =βt−β(η+μ)Γq(μ)∫0t(tβ−τβq)(μ−1)τβ(η+1)−1f(τ)dqτ  =β(1−q1/β)(1−q)μ−1∑k=0∞(qμ;q)k(q;q)kqk(η+1)f(tqk/β).

**Definition 2 .**

A *q*-analogue of the Kober fractional integral operator is given by (see, [20])

(27) Iqη,μ{f}(t):=(Iqη,μ,1{f}(t))=t−η−μΓq(μ)∫0t(t−τq)(μ−1)τηf(τ)dqτ,(μ>0;  η∈C;  0<q<1).

**Remark 3 .**

It is easy to see that

(28) $$\begin{array}{c} \Gamma_{q}\left(\mu \right)>0;\;\;{\left(q^{\mu };q\right)}_{k}>0, \end{array}$$

for all *μ* > 0 and *k* ∈ *N*_0_. If *f* : [0, *∞*)→[0, *∞*) is a continuous function, then we conclude that, under the given conditions in (26), each term in the series of generalized Erdélyi-Kober *q*-integral operator is nonnegative and thus

(29) $$\begin{array}{c} I_{q}^{\eta ,\mu ,\beta }\left\{f\right\}\left(t\right)\geq 0, \end{array}$$

for all *β*, *μ* > 0 and *η* ∈ *C*.

On the same way each term in the series of Kober *q*-integral operator (27) is also nonnegative and thus

(30) $$\begin{array}{c} I_{q}^{\eta ,\mu }\left\{f\right\}\left(t\right)\geq 0, \end{array}$$

for all *μ* > 0 and *η* ∈ *C*.

## 3. Inequalities Involving a Generalized Erdélyi-Kober Fractional *q*-Integral Operator for Synchronous Functions

This section begins by presenting two inequalities involving generalized Erdélyi-Kober *q*-integral operator (26) stated in Lemmas 4 and 5 below.

**Lemma 4 .**

Let 0 < *q* < 1, let *f* and *g* be two continuous and synchronous functions on [0, *∞*), and let *u*, *v* : [0, *∞*)→[0, *∞*) be continuous functions. Then, the following inequality holds true:

(31) Iqη,μ,β{u}(t)Iqη,μ,β{vfg}(t)  +Iqη,μ,β{v}(t)Iqη,μ,β{ufg}(t) ≥Iqη,μ,β{uf}(t)Iqη,μ,β{vg}(t)  +Iqη,μ,β{vf}(t)Iqη,μ,β{ug}(t),

for all *μ*, *β* > 0 and *η* ∈ *C*.

**Proof**

Let *f* and *g* be two continuous and synchronous functions on [0, *∞*). Then, for all *τ*, *ρ* ∈ (0, *t*) with *t* > 0, we have

(32) $$\begin{array}{c} \left(f\left(\tau \right)-f\left(\rho \right)\right)\left(g\left(\tau \right)-g\left(\rho \right)\right)\geq 0, \end{array}$$

or, equivalently,

(33) $$\begin{array}{c} f\left(\tau \right)g\left(\tau \right)+f\left(\rho \right)g\left(\rho \right)\geq f\left(\tau \right)g\left(\rho \right)+f\left(\rho \right)g\left(\tau \right). \end{array}$$

Now, multiplying both sides of (33) by (*βt* ^−*β*(*η*+*μ*)^/Γ_*q*_(*μ*))(*t* ^*β*^ − *τ* ^*β*^ *q*)^(*μ*−1)^ *τ* ^*β*(*η*+1)−1^ *u*(*τ*), integrating the resulting inequality with respect to *τ* from 0 to *t*, and using (26), we get

(34) Iqη,μ,β{ufg}(t)+f(ρ)g(ρ)Iqη,μ,β{u}(t)  ≥g(ρ)Iqη,μ,β{uf}(t)+f(ρ)Iqη,μ,β{ug}(t).

Next, multiplying both sides of (34) by (*βt* ^−*β*(*η*+*μ*)^/Γ_*q*_(*μ*))(*t* ^*β*^ − *ρ* ^*β*^ *q*)^(*μ*−1)^ *ρ* ^*β*(*η*+1)−1^ *v*(*ρ*), integrating the resulting inequality with respect to *ρ* from 0 to *t*, and using (26), we are led to the desired result (31).

**Lemma 5 .**

Let 0 < *q* < 1, let *f* and *g* be two continuous and synchronous functions on [0, *∞*), and let *u*, *v* : [0, *∞*)→[0, *∞*) be continuous functions. Then, the following inequality holds true:

(35) Iqζ,ν,δ{v}(t)Iqη,μ,β{ufg}(t)  +Iqζ,ν,δ{vfg}(t)Iqη,μ,β{u}(t) ≥Iqζ,ν,δ{vg}(t)Iqη,μ,β{uf}(t)  +Iqζ,ν,δ{vf}(t)Iqη,μ,β{ug}(t),

for all *μ*, *ν*, *β*, *δ* > 0 and *η*, *ζ* ∈ *C*.

**Proof**

Multiplying both sides of (34) by

(36) $$\begin{array}{c} \frac{\delta t^{-\delta (\zeta +\nu )}}{\Gamma_{q}\left(\nu \right)}{\left(t^{\delta }-\rho^{\delta }q\right)}^{\left(\nu -1\right)}\rho^{\delta (\zeta +1)-1}v\left(\rho \right), \end{array}$$

which remains nonnegative under the conditions in (35), integrating the resulting inequality with respect to *ρ* from 0 to *t*, and using (26), we get the desired result (35).

**Theorem 6 .**

Let 0 < *q* < 1, let *f* and *g* be two continuous and synchronous functions on [0, *∞*), and let *l*, *m*, *n* : [0, *∞*)→[0, *∞*) be continuous functions. Then, the following inequality holds true:

(37) 2Iqη,μ,β{l}(t)[Iqη,μ,β{m}(t)Iqη,μ,β{nfg}(t)+Iqη,μ,β{n}(t)Iqη,μ,β{mfg}(t)]  +2Iqη,μ,β{m}(t)Iqη,μ,β{n}(t)Iqη,μ,β{lfg}(t) ≥Iqη,μ,β{l}(t)[Iqη,μ,β{mf}(t)Iqη,μ,β{ng}(t)+Iqη,μ,β{nf}(t)Iqη,μ,β{mg}(t)]  +Iqη,μ,β{m}(t)[Iqη,μ,β{lf}(t)Iqη,μ,β{ng}(t)+Iqη,μ,β{nf}(t)Iqη,μ,β{lg}(t)]  +Iqη,μ,β{n}(t)[Iqη,μ,β{lf}(t)Iqη,μ,β{mg}(t)+Iqη,μ,β{mf}(t)Iqη,μ,β{lg}(t)],

for all *μ*, *β* > 0 and *η* ∈ *C*.

**Proof**

By setting *u* = *m* and *v* = *n* in Lemma 4, we get

(38) Iqη,μ,β{m}(t)Iqη,μ,β{nfg}(t)  +Iqη,μ,β{n}(t)Iqη,μ,β{mfg}(t) ≥Iqη,μ,β{mf}(t)Iqη,μ,β{ng}(t)  +Iqη,μ,β{nf}(t)Iqη,μ,β{mg}(t).

Since *I*_*q*_^*η*,*μ*,*β*^{*l*}(*t*) ≥ 0 under the given conditions, multiplying both sides of (38) by *I*_*q*_^*η*,*μ*,*β*^{*l*}(*t*), we have

(39) Iqη,μ,β{l}(t)[Iqη,μ,β{m}(t)Iqη,μ,β{nfg}(t)+Iqη,μ,β{n}(t)Iqη,μ,β{mfg}(t)] ≥Iqη,μ,β{l}(t)[Iqη,μ,β{mf}(t)Iqη,μ,β{ng}(t)+Iqη,μ,β{nf}(t)Iqη,μ,β{mg}(t)].

Similarly replacing *u*, *v* by *l*, *n* and *u*, *v* by *l*, *m*, respectively, in (31) and then multiplying both sides of the resulting inequalities by *I*_*q*_^*η*,*μ*,*β*^{*m*}(*t*) and *I*_*q*_^*η*,*μ*,*β*^{*n*}(*t*) both of which are nonnegative under the given assumptions, respectively, we get the following inequalities:

(40) Iqη,μ,β{m}(t)[Iqη,μ,β{l}(t)Iqη,μ,β{nfg}(t)+Iqη,μ,β{n}(t)Iqη,μ,β{lfg}(t)] ≥Iqη,μ,β{m}(t)[Iqη,μ,β{lf}(t)Iqη,μ,β{ng}(t)+Iqη,μ,β{nf}(t)Iqη,μ,β{lg}(t)],

(41) Iqη,μ,β{n}(t)[Iqη,μ,β{l}(t)Iqη,μ,β{mfg}(t)+Iqη,μ,β{m}(t)Iqη,μ,β{lfg}(t)] ≥Iqη,μ,β{n}(t)[Iqη,μ,β{lf}(t)Iqη,μ,β{mg}(t)+Iqη,μ,β{mf}(t)Iqη,μ,β{lg}(t)].

Finally, by adding (39), (40), and (41), side by side, we arrive at the desired result (37).

**Theorem 7 .**

Let 0 < *q* < 1, let *f* and *g* be two continuous and synchronous functions on [0, *∞*),* and let l*, *m*, *n* : [0, *∞*)→[0, *∞*) be continuous functions. Then, the following inequality holds true:

(42) Iqη,μ,β{l}(t)[2Iqη,μ,β{m}(t)Iqζ,ν,δ{nfg}(t)+Iqη,μ,β{n}(t)Iqζ,ν,δ{mfg}(t)+Iqζ,ν,δ{n}(t)Iqη,μ,β{mfg}(t)]  +Iqη,μ,β{lfg}(t)[Iqη,μ,β{m}(t)Iqζ,ν,δ{n}(t)+Iqη,μ,β{n}(t)Iqζ,ν,δ{m}(t)] ≥Iqη,μ,β{l}(t)[Iqη,μ,β{mf}(t)Iqζ,ν,δ{ng}(t)+Iqη,μ,β{mg}(t)Iqζ,ν,δ{nf}(t)]  +Iqη,μ,β{m}(t)[Iqη,μ,β{lf}(t)Iqζ,ν,δ{ng}(t)+Iqη,μ,β{lg}(t)Iqζ,ν,δ{nf}(t)]  +Iqη,μ,β{n}(t)[Iqη,μ,β{lf}(t)Iqζ,ν,δ{mg}(t)+Iqη,μ,β{lg}(t)Iqζ,ν,δ{mf}(t)],

for all *μ*, *ν*, *β*, *δ* > 0 and *η*, *ζ* ∈ *C*.

**Proof**

Setting *u* = *m* and *v* = *n* in (35), we have

(43) Iqζ,ν,δ{n}(t)Iqη,μ,β{mfg}(t)  +Iqζ,ν,δ{nfg}(t)Iqη,μ,β{m}(t) ≥Iqζ,ν,δ{ng}(t)Iqη,μ,β{mf}(t)  +Iqζ,ν,δ{nf}(t)Iqη,μ,β{mg}(t).

Multiplying both sides of (43) by *I*_*q*_^*η*,*μ*,*β*^{*l*}(*t*), after a little simplification, we get

(44) Iqη,μ,β{l}(t)[Iqζ,ν,δ{n}(t)Iqη,μ,β{mfg}(t)+Iqζ,ν,δ{nfg}(t)Iqη,μ,β{m}(t)] ≥Iqη,μ,β{l}(t)[Iqζ,ν,δ{ng}(t)Iqη,μ,β{mf}(t)+Iqζ,ν,δ{nf}(t)Iqη,μ,β{mg}(t)].

Now, by replacing *u*, *v* by *l*, *n* and *u*, *v* by *l*, *m* in (35), respectively, and then multiplying both sides of the resulting inequalities by *I*_*q*_^*η*,*μ*,*β*^{*m*}(*t*) and *I*_*q*_^*η*,*μ*,*β*^{*n*}(*t*), respectively, we get the following two inequalities:

(45) Iqη,μ,β{m}(t)[Iqζ,ν,δ{n}(t)Iqη,μ,β{lfg}(t)+Iqζ,ν,δ{nfg}(t)Iqη,μ,β{l}(t)] ≥Iqη,μ,β{m}(t)[Iqζ,ν,δ{ng}(t)Iqη,μ,β{lf}(t)+Iqζ,ν,δ{nf}(t)Iqη,μ,β{lg}(t)],Iqη,μ,β{n}(t)[Iqζ,ν,δ{m}(t)Iqη,μ,β{lfg}(t)+Iqζ,ν,δ{mfg}(t)Iqη,μ,β{l}(t)] ≥Iqη,μ,β{n}(t)[Iqζ,ν,δ{mg}(t)Iqη,μ,β{lf}(t)+Iqζ,ν,δ{mf}(t)Iqη,μ,β{lg}(t)].

Finally, we find that the inequality (42) follows by adding the inequalities (44) and (45), side by side.

**Remark 8 .**

It may be noted that inequalities (37) and (42) in Theorems 6 and 7, respectively, are reversed if the functions are asynchronous on [0, *∞*). The special case of (42) in Theorem 7 when *β* = *δ*, *η* = *ζ*, and *μ* = *ν* is easily seen to yield inequality (37) in Theorem 6.

**Remark 9 .**

We remark further that we can present a large number of special cases of our main inequalities in Theorems 6 and 7. Here, we give only two examples: setting *β* = 1 in (37) and *β* = *δ* = 1 in (42), we obtain interesting inequalities involving Erdélyi-Kober fractional integral operator.

**Corollary 10 .**

Let 0 < *q* < 1, let *f* and *g* be two continuous and synchronous functions on [0, *∞*), and let *l*, *m*, *n* : [0, *∞*)→[0, *∞*) be continuous functions. Then, the following inequality holds true:

(46) 2Iqη,μ{l}(t)[Iqη,μ{m}(t)Iqη,μ{nfg}(t)+Iqη,μ{n}(t)Iqη,μ{mfg}(t)]  +2Iqη,μ{m}(t)Iqη,μ{n}(t)Iqη,μ{lfg}(t) ≥Iqη,μ{l}(t)[Iqη,μ{mf}(t)Iqη,μ{ng}(t)+Iqη,μ{nf}(t)Iqη,μ{mg}(t)]  +Iqη,μ{m}(t)[Iqη,μ{lf}(t)Iqη,μ{ng}(t)+Iqη,μ{nf}(t)Iqη,μ{lg}(t)]  +Iqη,μ{n}(t)[Iqη,μ{lf}(t)Iqη,μ{mg}(t)+Iqη,μ{mf}(t)Iqη,μ{lg}(t)],

for all *μ* > 0 and *η* ∈ *C*.

**Corollary 11 .**

Let 0 < *q* < 1, let *f* and *g* be two continuous and synchronous functions on [0, *∞*), and let *l*, *m*, *n* : [0, *∞*)→[0, *∞*) be continuous functions. Then, the following inequality holds true:

(47) Iqη,μ{l}(t)[2Iqη,μ{m}(t)Iqζ,ν{nfg}(t)+Iqη,μ{n}(t)Iqζ,ν{mfg}(t)+Iqζ,ν{n}(t)Iqη,μ{mfg}(t)]  +Iqη,μ{lfg}(t)[Iqη,μ{m}(t)Iqζ,ν{n}(t)+Iqη,μ{n}(t)Iqζ,ν{m}(t)] ≥Iqη,μ{l}(t)[Iqη,μ{mf}(t)Iqζ,ν{ng}(t)+Iqη,μ{mg}(t)Iqζ,ν{nf}(t)]  +Iqη,μ{m}(t)[Iqη,μ{lf}(t)Iqζ,ν{ng}(t)+Iqη,μ{lg}(t)Iqζ,ν{nf}(t)]  +Iqη,μ{n}(t)[Iqη,μ{lf}(t)Iqγ,δ{mg}(t)+Iqη,μ{lg}(t)Iqζ,ν{mf}(t)],

for all *μ*, *ν* > 0 and *η*, *ζ* ∈ *C*.

**Remark 12 .**

If we take *η* = 0 and *β* = 1 in Theorem 6 and *η* = *ζ* = 0 and *β* = *δ* = 1 in Theorem 7, then we obtain the known results due to Dahmani [21].

## 4. Inequalities Involving a Generalized Erdélyi-Kober Fractional *q*-Integral Operator for Bounded Functions

In this section we obtain some new inequalities involving Erdélyi-Kober fractional *q*-integral operator in the case where the functions are bounded by integrable functions and are not necessary increasing or decreasing as are the synchronous functions.

**Theorem 13 .**

Let 0 < *q* < 1, let *f* be an integrable function on [0, *∞*), and let *u*, *v* : [0, *∞*)→[0, *∞*) be continuous functions. Assume the following.

 (*H*_1_)
There exist two integrable functions *φ*_1_, *φ*_2_ on [0, *∞*) such that
  (48) $$\begin{array}{c} \varphi_{1}\left(t\right)\leq f\left(t\right)\leq \varphi_{2}\left(t\right),\;\forall t\in \left[0,\infty \right). \end{array}$$

Then, for *t* > 0, *μ*, *β* > 0, and *η* ∈ *C*, we have

(49) Iqη,μ,β{uφ2}(t)Iqη,μ,β{vf}(t)  +Iqη,μ,β{uf}(t)Iqη,μ,β{vφ1}(t) ≥Iqη,μ,β{uφ2}(t)Iqη,μ,β{vφ1}(t)  +Iqη,μ,β{uf}(t)Iqη,μ,β{vf}(t).

**Proof**

From (*H*_1_), for all *τ* ≥ 0 and *ρ* ≥ 0, we have

(50) $$\begin{array}{c} \left(\varphi_{2}\left(\tau \right)-f\left(\tau \right)\right)\left(f\left(\rho \right)-\varphi_{1}\left(\rho \right)\right)\geq 0. \end{array}$$

Therefore,

(51) φ2(τ)f(ρ)+φ1(ρ)f(τ)  ≥φ1(ρ)φ2(τ)+f(τ)f(ρ).

Multiplying both sides of (51) by (*βt* ^−*β*(*η*+*μ*)^/Γ_*q*_(*μ*))(*t* ^*β*^ − *τ* ^*β*^ *q*)^(*μ*−1)^ *τ* ^*β*(*η*+1)−1^ *u*(*τ*), *τ* ∈ (0, *t*), and integrating both sides with respect to *τ* on (0, *t*), we obtain

(52) Iqη,μ,β{uφ2}(t)f(ρ)+Iqη,μ,β{uf}(t)φ1(ρ)  ≥Iqη,μ,β{uφ2}(t)φ1(ρ)+Iqη,μ,β{uf}(t)f(ρ).

Multiplying both sides of (52) by (*βt* ^−*β*(*η*+*μ*)^/Γ_*q*_(*μ*))(*t* ^*β*^ − *ρ* ^*β*^ *q*)^(*μ*−1)^ *ρ* ^*β*(*η*+1)−1^ *v*(*ρ*), *ρ* ∈ (0, *t*), and integrating both sides with respect to *ρ* on (0, *t*), we get inequality (49) as requested. This completes the proof.

As special cases of Theorems 13, we obtain the following results.

**Corollary 14 .**

Let 0 < *q* < 1, let *f* be an integrable function on [0, *∞*) satisfying *m* ≤ *f*(*t*) ≤ *M*, for all *t* ∈ [0, *∞*), let *u*, *v* : [0, *∞*)→[0, *∞*) be continuous functions, and let  *m*, *M* ∈ *R*. Then, for *t* > 0, *μ*, *β* > 0, and *η* ∈ *C*, we have

(53) MIqη,μ,β{u}(t)Iqη,μ,β{vf}(t)  +mIqη,μ,β{uf}(t)Iqη,μ,β{v}(t) ≥mMIqη,μ,β{u}(t)Iqη,μ,β{v}(t)  +Iqη,μ,β{uf}(t)Iqη,μ,β{vf}(t).

**Corollary 15 .**

Let 0 < *q* < 1, let *f* be an integrable function on [1, *∞*), and let *u*, *v* : [0, *∞*)→[0, *∞*) be continuous functions. Assume that there exists an integrable function *φ*(*t*) on [0, *∞*) and a constant *M* > 0 such that

(54) $$\begin{array}{c} \varphi \left(t\right)-M\leq f\left(t\right)\leq \varphi \left(t\right)+M, \end{array}$$

for all *t* > 0, *μ*, *β* > 0, and *η* ∈ *C*; we have

(55) Iqη,μ,β{uφ}(t)Iqη,μ,β{vf}(t)  +Iqη,μ,β{uf}(t)Iqη,μ,β{vφ}(t)  +MIqη,μ,β{u}(t)Iqη,μ,β{vf}(t)  +MIqη,μ,β{v}(t)Iqη,μ,β{uφ}(t)  +M2Iqη,μ,β{u}(t)Iqη,μ,β{v}(t) ≥Iqη,μ,β{uφ}(t)Iqη,μ,β{vφ}(t)  +Iqη,μ,β{uf}(t)Iqη,μ,β{vf}(t)  +MIqη,μ,β{u}(t)Iqη,μ,β{vφ}(t)  +MIqη,μ,β{uf}(t)Iqη,μ,β{v}(t).

**Theorem 16 .**

Let 0 < *q* < 1, let *f* be an integrable function on [0, *∞*), let *u*, *v* : [0, *∞*)→[0, *∞*) be continuous functions, and let *θ*_1_, *θ*_2_ > 0 satisfying 1/*θ*_1_ + 1/*θ*_2_ = 1. Suppose that (*H*_1_) holds. Then, for *t* > 0, *μ*, *β* > 0, and *η* ∈ *C*, we have

(56) 1θ1Iqη,μ,β{v}(t)Iqη,μ,β{u(φ2−f)θ1}(t)  +1θ2Iqη,μ,β{u}(t)Iqη,μ,β{v(f−φ1)θ2}(t)  +Iqη,μ,β{uφ2}(t)Iqη,μ,β{vφ1}(t)  +Iqη,μ,β{uf}(t)Iqη,μ,β{vf}(t) ≥Iqη,μ,β{uφ2}(t)Iqη,μ,β{vf}(t)  +Iqη,μ,β{uf}(t)Iqη,μ,β{vφ1}(t).

**Proof**

According to the well-known Young inequality [3]

(57) 1θ1xθ1+1θ2yθ2≥xy, ∀x,y≥0,θ1,θ2>0, 1θ1+1θ2=1,

and setting *x* = *φ*_2_(*τ*) − *f*(*τ*) and *y* = *f*(*ρ*) − *φ*_1_(*ρ*), *τ*, *ρ* ≥ 0, we have

(58) 1θ1(φ2(τ)−f(τ))θ1+1θ2(f(ρ)−φ1(ρ))θ2  ≥(φ2(τ)−f(τ))(f(ρ)−φ1(ρ)).

Multiplying both sides of (58) by

(59) β2t−2β(η+μ)Γq2(μ)(tβ−τβq)(μ−1)(tβ−ρβq)(μ−1)  ×(τρ)β(η+1)−1u(τ)v(ρ),

for *τ*, *ρ* ∈ (0, *t*), and integrating with respect to *τ* and *ρ* from 0 to *t*, we deduce the desired result in (56).

**Corollary 17 .**

Let 0 < *q* < 1, let *f* be an integrable function on [0, *∞*) satisfying *m* ≤ *f*(*t*) ≤ *M*, for all *t* ∈ [0, *∞*), let *u*, *v* : [0, *∞*)→[0, *∞*) be continuous functions, and let *m*, *M* ∈ *R*. Then, for *t* > 0, *μ*, *β* > 0, and *η* ∈ *C*, we have

(60) (m+M)2Iqη,μ,β{u}(t)Iqη,μ,β{v}(t)  +2Iqη,μ,β{uf}(t)Iqη,μ,β{vf}(t)  +Iqη,μ,β{vf2}(t)(Iqη,μ,β{u}(t)+Iqη,μ,β{v}(t)) ≥2(m+M)(Iqη,μ,β{uf}(t)Iqη,μ,β{v}(t)+ Iqη,μ,β{u}(t)Iqη,μ,β{vf}(t)).

**Theorem 18 .**

Let 0 < *q* < 1, let *f* be an integrable function on [0, *∞*), let *u*, *v* : [0, *∞*)→[0, *∞*) be continuous functions, and let *θ*_1_, *θ*_2_ > 0 satisfying *θ*_1_ + *θ*_2_ = 1. In addition, suppose that (*H*_1_) holds. Then, for *t* > 0, *μ*, *β* > 0, and *η* ∈ *C*, we have

(61) θ1Iqη,μ,β{uφ2}(t)Iqη,μ,β{v}(t)  +θ2Iqη,μ,β{u}(t)Iqη,μ,β{vf}(t) ≥θ1Iqη,μ,β{uf}(t)Iqη,μ,β{v}(t)  +θ2Iqη,μ,β{u}(t)Iqη,μ,β{vφ1}(t)  +Iqη,μ,β{u(φ2−f)θ1}(t)Iqη,μ,β{v(f−φ1)θ2}(t).

**Proof**

From the well-known Weighted AM-GM inequality [3]

(62) θ1x+θ2y≥xθ1yθ2, ∀x,y≥0,  θ1,θ2>0, θ1+θ2=1,

by setting *x* = *φ*_2_(*τ*) − *f*(*τ*) and *y* = *f*(*ρ*) − *φ*_1_(*ρ*), *τ*, *ρ* > 1, we have

(63) θ1(φ2(τ)−f(τ))+θ2(f(ρ)−φ1(ρ))  ≥(φ2(τ)−f(τ))θ1(f(ρ)−φ1(ρ))θ2.

Multiplying both sides of (63) by

(64) β2t−2β(η+μ)Γq2(μ)(tβ−τβq)(μ−1)(tβ−ρβq)(μ−1)  × (τρ)β(η+1)−1u(τ)v(ρ),

for *τ*, *ρ* ∈ (0, *t*), and integrating with respect to *τ* and *ρ* from 0 to *t*, we deduce inequality (61).

**Corollary 19 .**

Let 0 < *q* < 1, let *f* be an integrable function on [0, *∞*) satisfying *m* ≤ *f*(*t*) ≤ *M*, for all *t* ∈ [0, *∞*), let *u*, *v* : [0, *∞*)→[0, *∞*) be continuous functions, and let *m*, *M* ∈ *R*. Then, for *t* > 0, *μ*, *β* > 0, and *η* ∈ *C*, we have

(65) (M−m)Iqη,μ,β{u}(t)Iqη,μ,β{v}(t)+Iqη,μ,β{u}(t)Iqη,μ,β{vf}(t) ≥Iqη,μ,β{uf}(t)Iqη,μ,β{v}(t)  +2Iqη,μ,β{uM−f}(t)Iqη,μ,β{vf−m}(t).

**Lemma 20 (see [22]).**

Assume that *a* ≥ 0, *p* ≥ *q* ≥ 0, and *p* ≠ 0. Then,

(66) $$\begin{array}{c} a^{q/p}\leq \left(\frac{q}{p}k^{\left(q-p\right)/p}a+\frac{p-q}{p}k^{q/p}\right),\;for\;\;any\;\;k>0. \end{array}$$

**Theorem 21 .**

Let 0 < *q* < 1, let *f* be an integrable function on [0, *∞*), let *u* : [0, *∞*)→[0, *∞*) be a continuous function, and let constants *p* ≥ *q* ≥ 0, *p* ≠ 0. In addition, assume that (*H*_1_) holds. Then, for any *k* > 0, *t* > 0, *μ*, *β* > 0, and *η* ∈ *C*, the following two inequalities hold:

(67) (i)  Iqη,μ,β{u(φ2−f)q/p}(t)+qpk(q−p)/pIqη,μ,β{uf}(t)  ≤qpk(q−p)/pIqη,μ,β{uφ2}(t)+p−qpkq/pIqη,μ,β{u}(t),

(68) (ii)  Iqη,μ,β{u(f−φ1)q/p}(t)+qpk(q−p)/pIqη,μ,β{uφ1}(t)  ≤qpk(q−p)/pIqη,μ,β{uf}(t)+p−qpkq/pIqη,μ,β{u}(t).

**Proof**

By condition (*H*_1_) and Lemma 20, for *p* ≥ *q* ≥ 0, *p* ≠ 0, it follows that

(69) $$\begin{array}{c} {\left(\varphi_{2}\left(\tau \right)-f\left(\tau \right)\right)}^{q/p}\leq \frac{q}{p}k^{(q-p)/p}\left(\varphi_{2}\left(\tau \right)-f\left(\tau \right)\right)+\frac{p-q}{p}k^{q/p}, \end{array}$$

for any *k* > 0. Multiplying both sides of (69) by (*βt* ^−*β*(*η*+*μ*)^/Γ_*q*_(*μ*))(*t* ^*β*^ − *τ* ^*β*^ *q*)^(*μ*−1)^ *τ* ^*β*(*η*+1)−1^ *u*(*τ*), *τ* ∈ (0, *t*), and integrating the resulting identity with respect to *τ* from 0 to *t*, one has inequality (*i*). Inequality (*ii*) is proved by setting *a* = *f*(*τ*) − *φ*_1_(*τ*) in Lemma 20.

**Corollary 22 .**

Let 0 < *q* < 1, let *f* be an integrable function on [0, *∞*) satisfying *m* ≤ *f*(*t*) ≤ *M*, for all *t* ∈ [0, *∞*), let *u*, *v* : [0, *∞*)→[0, *∞*) be continuous functions, and let *m*, *M* ∈ *R*. Then, for *t* > 0, *μ*, *β* > 0, and *η* ∈ *C*, we have

(70) (i)  2Iqη,μ,β{uM−f}(t)+Iqη,μ,β{uf}(t)  ≤(M+1)Iqη,μ,β{u}(t),(ii)  2Iqη,μ,β{uf−m}(t)+(m−1)Iqη,μ,β{u}(t)  ≤Iqη,μ,β{uf}(t).

**Theorem 23 .**

Let 0 < *q* < 1, let *f* and *g* be two integrable functions on [0, *∞*), and let *u*, *v* : [0, *∞*)→[0, *∞*) be continuous functions. Suppose that (*H*_1_) holds and moreover we assume the following.

 (*H*_2_) There exist *ψ*_1_ and *ψ*_2_ integrable functions on [0, *∞*) such that
  (71) $$\begin{array}{c} \psi_{1}\left(t\right)\leq g\left(t\right)\leq \psi_{2}\left(t\right)\;\forall t\in \left[0,\infty \right). \end{array}$$

Then, for *t* > 0, *μ*, *β* > 0, and *η* ∈ *C*, the following inequalities hold:

(72) (i)  Iqη,μ,β{uφ2}(t)Iqη,μ,β{vg}(t)    +Iqη,μ,β{uf}(t)Iqη,μ,β{vψ1}(t)   ≥Iqη,μ,β{uφ2}(t)Iqη,μ,β{vψ1}(t)    +Iqη,μ,β{uf}(t)Iqη,μ,β{vg}(t),(ii)  Iqη,μ,β{uψ2}(t)Iqη,μ,β{vf}(t)    +Iqη,μ,β{ug}(t)Iqη,μ,β{vφ1}(t)   ≥Iqη,μ,β{uψ2}(t)Iqη,μ,β{vφ1}(t)    +Iqη,μ,β{ug}(t)Iqη,μ,β{vf}(t),(iii)  Iqη,μ,β{uφ2}(t)Iqη,μ,β{vψ2}(t)    +Iqη,μ,β{uf}(t)Iqη,μ,β{vg}(t)   ≥Iqη,μ,β{uφ2}(t)Iqη,μ,β{vg}(t)    +Iqη,μ,β{uf}(t)Iqη,μ,β{vψ2}(t),(iv)  Iqη,μ,β{uφ1}(t)Iqη,μ,β{vψ1}(t)    +Iqη,μ,β{uf}(t)Iqη,μ,β{vg}(t)   ≥Iqη,μ,β{uφ1}(t)Iqη,μ,β{vg}(t)    +Iqη,μ,β{uf}(t)Iqη,μ,β{vψ1}(t).

**Proof**

To prove (*i*), from (*H*_1_) and (*H*_2_), we have for *t* ∈ [0, *∞*) that

(73) $$\begin{array}{c} \left(\varphi_{2}\left(\tau \right)-f\left(\tau \right)\right)\left(g\left(\rho \right)-\psi_{1}\left(\rho \right)\right)\geq 0. \end{array}$$

Therefore,

(74) φ2(τ)g(ρ)+ψ1(ρ)f(τ)≥ψ1(ρ)φ2(τ) +f(τ)g(ρ).

Multiplying both sides of (74) by (*βt* ^−*β*(*η*+*μ*)^/Γ_*q*_(*μ*))(*t* ^*β*^ − *τ* ^*β*^ *q*)^(*μ*−1)^ *τ* ^*β*(*η*+1)−1^ *u*(*τ*), *τ* ∈ (0, *t*), and integrating both sides with respect to *τ* on (0, *t*), we obtain

(75) g(ρ)Iqη,μ,β{uφ2}(t)+ψ1(ρ)Iqη,μ,β{uf}(t)  ≥ψ1(ρ)Iqη,μ,β{uφ2}(t)+g(ρ)Iqη,μ,β{uf}(t).

Multiplying both sides of (75) by (*βt* ^−*β*(*η*+*μ*)^/Γ_*q*_(*μ*))(*t* ^*β*^ − *ρ* ^*β*^ *q*)^(*μ*−1)^ *ρ* ^*β*(*η*+1)−1^ *v*(*ρ*), *ρ* ∈ (0, *t*), and integrating both sides with respect to *ρ* on (0, *t*), we get the desired inequality (*i*).

To prove (*ii*)–(*iv*), we use the following inequalities:

(76)  (ii)  (ψ2(τ)−g(τ))(f(ρ)−φ1(ρ))≥0, (iii)  (φ2(τ)−f(τ))(g(ρ)−ψ2(ρ))≤0, (iv)  (φ1(τ)−f(τ))(g(ρ)−ψ1(ρ))≤0.

**Theorem 24 .**

Let *f* and *g* be two integrable functions on [0, *∞*), let *u*, *v* : [0, *∞*)→[0, *∞*) be continuous functions, and let *θ*_1_, *θ*_2_ > 0 satisfying 1/*θ*_1_ + 1/*θ*_2_ = 1. Suppose that (*H*_1_) and (*H*_2_) hold. Then, for *t* > 0, *μ*, *β* > 0, and *η* ∈ *C*, the following inequalities hold:

(77) (i)  1θ1Iqη,μ,β{u(φ2−f)θ1}(t)Iqη,μ,β{v}(t)    +1θ2Iqη,μ,β{v(ψ2−g)θ2}(t)Iqη,μ,β{u}(t)    +Iqη,μ,β{uφ2}(t)Iqη,μ,β{vg}(t)    +Iqη,μ,β{uf}(t)Iqη,μ,β{vψ2}(t)   ≥Iqη,μ,β{uφ2}(t)Iqη,μ,β{vψ2}(t)    +Iqη,μ,β{uf}(t)Iqη,μ,β{vg}(t),(ii)  1θ1Iqη,μ,β{u(φ2−f)θ1}(t)Iqη,μ,β{v(ψ2−g)θ1}(t)    +1θ2Iqη,μ,β{u(ψ2−g)θ2}(t)Iqη,μ,β{v(φ2−f)θ2}(t)   ≥Iqη,μ,β{u(φ2−f)(ψ2−g)}(t)    ×Iqη,μ,β{v(ψ2−g)(φ2−f)}(t),(iii) 1θ1Iqη,μ,β{u(f−φ1)θ1}(t)Iqη,μ,β{v}(t)     +1θ2Iqη,μ,β{v(g−ψ1)θ2}(t)Iqη,μ,β{u}(t)    +Iqη,μ,β{uf}(t)Iqη,μ,β{vψ1}(t)    +Iqη,μ,β{uφ1}(t)Iqη,μ,β{vg}(t)   ≥Iqη,μ,β{uf}(t)Iqη,μ,β{vg}(t)    +Iqη,μ,β{uφ1}(t)Iqη,μ,β{vψ1}(t),(iv)  1θ1Iqη,μ,β{u(f−φ1)θ1}(t)Iqη,μ,β{v(g−ψ1)θ1}(t)    +1θ2Iqη,μ,β{u(g−ψ1)θ2}(t)Iqη,μ,β{v(f−φ1)θ2}(t)   ≥Iqη,μ,β{u(f−φ1)(g−ψ1)}(t)    ×Iqη,μ,β{v(g−ψ1)(f−φ1)}(t).

**Proof**

The inequalities (*i*)–(*iv*) can be proved by choosing the parameters in the Young inequality [3]:

(78) (i)  x=φ2(τ)−f(τ),  y=ψ2(ρ)−g(ρ),(ii)  x=(φ2(τ)−f(τ))(ψ2(ρ)−g(ρ)),y=(ψ2(τ)−g(τ))(φ2(ρ)−f(ρ)),(iii)  x=f(τ)−φ1(τ),  y=g(ρ)−ψ1(ρ),(iv)  x=(f(τ)−φ1(τ))(g(ρ)−ψ1(ρ)),y=(g(τ)−ψ1(τ))(f(ρ)−φ1(ρ)).

**Theorem 25 .**

Let *f* and *g* be two integrable functions on [0, *∞*), let *u*, *v* : [0, *∞*)→[0, *∞*) be continuous functions, and let *θ*_1_, *θ*_2_ > 0 satisfying *θ*_1_ + *θ*_2_ = 1. Suppose that (*H*_1_) and (*H*_2_) hold. Then, for *t* > 0, *μ*, *β* > 0, and *η* ∈ *C*, the following inequalities hold:

(79) (i)  θ1Iqη,μ,β{uφ2}(t)Iqη,μ,β{v}(t)    +θ2Iqη,μ,β{vψ2}(t)Iqη,μ,β{u}(t)   ≥θ1Iqη,μ,β{uf}(t)Iqη,μ,β{v}(t)    +θ2Iqη,μ,β{vg}(t)Iqη,μ,β{u}(t)    +Iqη,μ,β{u(φ2−f)θ1}(t)Iqη,μ,β{v(ψ2−g)θ2}(t),(ii)  θ1Iqη,μ,β{uφ2}(t)Iqη,μ,β{vψ2}(t)    +θ1Iqη,μ,β{uf}(t)Iqη,μ,β{vg}(t)    +θ2Iqη,μ,β{uψ2}(t)Iqη,μ,β{vφ2}(t)    +θ2Iqη,μ,β{ug}(t)Iqη,μ,β{vf}(t)   ≥θ1Iqη,μ,β{uφ2}(t)Iqη,μ,β{vg}(t)    +θ1Iqη,μ,β{uf}(t)Iqη,μ,β{vψ2}(t)    +θ2Iqη,μ,β{uψ2}(t)Iqη,μ,β{vf}(t)    +θ2Iqη,μ,β{ug}(t)Iqη,μ,β{vφ2}(t)    +Iqη,μ,β{u(φ2−f)θ1(ψ2−g)θ2}(t)    ×Iqη,μ,β{v(ψ2−g)θ1(φ2−f)θ2}(t),(iii)  θ1Iqη,μ,β{uf}(t)Iqη,μ,β{v}(t)    +θ2Iqη,μ,β{vg}(t)Iqη,μ,β{u}(t)   ≥θ1Iqη,μ,β{uφ1}(t)Iqη,μ,β{v}(t)    +θ2Iqη,μ,β{vψ1}(t)Iqη,μ,β{u}(t)    +Iqη,μ,β{u(f−φ1)θ1}(t)Iqη,μ,β{v(g−ψ1)θ2}(t),(iv)  θ1Iqη,μ,β{uf}(t)Iqη,μ,β{vg}(t)    +θ1Iqη,μ,β{uφ1}(t)Iqη,μ,β{vψ1}(t)    +θ2Iqη,μ,β{ug}(t)Iqη,μ,β{vf}(t)    +θ2Iqη,μ,β{uψ1}(t)Iqη,μ,β{vφ1}(t)   ≥θ1Iqη,μ,β{uf}(t)Iqη,μ,β{vψ1}(t)    +θ1Iqη,μ,β{uφ1}(t)Iqη,μ,β{vg}(t)    +θ2Iqη,μ,β{ug}(t)Iqη,μ,β{vφ1}(t)    +θ2Iqη,μ,β{uψ1}(t)Iqη,μ,β{vf}(t)    +Iqη,μ,β{u(f−φ1)θ1(g−ψ1)θ2}(t)    ×Iqη,μ,β{v(g−ψ1)θ1(f−φ1)θ2}(t).

**Proof**

The inequalities (*i*)–(*iv*) can be proved by choosing the parameters in the Weighted AM-GM [3]:

(80) (i)  x=φ2(τ)−f(τ),  y=ψ2(ρ)−g(ρ),(ii)  x=(φ2(τ)−f(τ))(ψ2(ρ)−g(ρ)),y=(ψ2(τ)−g(τ))(φ2(ρ)−f(ρ)),(iii)  x=f(τ)−φ1(τ),  y=g(ρ)−ψ1(ρ),(iv)  x=(f(τ)−φ1(τ))(g(ρ)−ψ1(ρ)),y=(g(τ)−ψ1(τ))(f(ρ)−φ1(ρ)).

**Theorem 26 .**

Let *f* and *g* be two integrable functions on [0, *∞*), let *u*, *v* : [0, *∞*)→[0, *∞*) be continuous functions, and let constants *p* ≥ *q* ≥ 0, *p* ≠ 0. Assume that (*H*_1_) and (*H*_2_) hold. Then, for any *k* > 0, *t* > 0, *μ*, *β* > 0, and *η* ∈ *C*, the following inequalities hold:

(81) (i)  Iqη,μ,β{u(φ2−f)q/p(ψ2−g)q/p}(t)   +qpk(q−p)/pIqη,μ,β{uφ2g}(t)   +qpk(q−p)/pIqη,μ,β{ufψ2}(t)  ≤qpk(q−p)/pIqη,μ,β{uφ2ψ2}(t)   +qpk(q−p)/pIqη,μ,β{ufg}(t)   +p−qpkq/pIqη,μ,β{u}(t),(ii)  Iqη,μ,β{u(φ2−f)q/p}(t)Iqη,μ,β{v(ψ2−g)q/p}(t)   +qpk(q−p)/pIqη,μ,β{uφ2}(t)Iqη,μ,β{vg}(t)   +qpk(q−p)/pIqη,μ,β{uf}(t)Iqη,μ,β{vψ2}(t)  ≤qpk(q−p)/pIqη,μ,β{uφ2}(t)Iqη,μ,β{vψ2}(t)   +qpk(q−p)/pIqη,μ,β{uf}(t)Iqη,μ,β{vg}(t)g(t)   +p−qpkq/pIqη,μ,β{u}(t)Iqη,μ,β{v}(t),(iii)  Iqη,μ,β{u(f−φ1)q/p(g−ψ1)q/p}(t)   +qpk(q−p)/pIqη,μ,β{uψ1f}(t)   +qpk(q−p)/pIqη,μ,β{uφ1g}(t)  ≤qpk(q−p)/pIqη,μ,β{ufg}(t)   +qpk(q−p)/pIqη,μ,β{uφ1ψ1}(t)   +p−qpkq/pIqη,μ,β{u}(t),(iv)  Iqη,μ,β{u(f−φ1)q/p}(t)Iqη,μ,β{v(g−ψ1)q/p}(t)   +qpk(q−p)/pIqη,μ,β{uf}(t)Iqη,μ,β{vψ1}(t)   +qpk(q−p)/pIqη,μ,β{uφ1}(t)Iqη,μ,β{vg}(t)  ≤qpk(q−p)/pIqη,μ,β{uf}(t)Iqη,μ,β{vg}(t)   +qpk(q−p)/pIqη,μ,β{uφ1}(t)Iqη,μ,β{vψ1}(t)   +p−qpkq/pIqη,μ,β{u}(t)Iqη,μ,β{v}(t).

**Proof**

The inequalities (*i*)–(*iv*) can be proved by choosing the parameters in Lemma 20:

(82)  (i)  a=(φ2(τ)−f(τ))(ψ2(τ)−g(τ)), (ii)  a=(φ2(τ)−f(τ))(ψ2(ρ)−g(ρ)), (iii)  a=(f(τ)−φ1(τ))(g(τ)−ψ1(τ)), (iv)  a=(f(τ)−φ1(τ))(g(ρ)−ψ1(ρ)).

## 5. Concluding Remark

We conclude our present investigation with the remark that the results derived in this paper are general in character and give some contributions to the theory of *q*-integral inequalities and fractional calculus. Moreover, they are expected to find some applications for establishing uniqueness of solutions in fractional boundary value problems and in fractional partial differential equations. In last, use of the generalized Erdélyi-Kober fractional *q*-integral operator due to Gaulué is the advantage of our results because after setting suitable parameter values in our main results, we get known results established by number of authors.
